# Bridging the Gap between Preclinical and Clinical Microbicide Trials: Blind Evaluation of Candidate Gels in Murine Models of Efficacy and Safety

**DOI:** 10.1371/journal.pone.0027675

**Published:** 2011-11-11

**Authors:** Theodore J. Segarra, Esra Fakioglu, Natalia Cheshenko, Sarah S. Wilson, Pedro M. M. Mesquita, Gustavo F. Doncel, Betsy C. Herold

**Affiliations:** 1 Department of Pediatrics and Microbiology-Immunology, Albert Einstein College of Medicine, Bronx, New York, United States of America; 2 CONRAD, Department of Obstetrics and Gynecology, Eastern Virginia Medical School, Norfolk, Virginia, United States of America; Columbia University, United States of America

## Abstract

**Background:**

Despite significant protection in preclinical studies, cellulose sulfate (CS) failed to protect women against HIV-1/2 and was associated with a trend toward increased HIV-1 acquisition in one of the clinical trials. These results highlight the need for preclinical tests more predictive of clinical outcomes. The objective of this study was to test coded vaginal gels, including CS, in murine models of safety and efficacy to determine the models' utility for evaluating future products.

**Methods:**

Four coded formulations, including 6% CS, 2% PRO 2000 and two placebo gels, were administered intravaginally to medroxyprogesterone-treated mice and their ability to prevent genital herpes (efficacy) or to alter the susceptibility to low dose HSV challenge (safety) was determined. Nonoyxnol-9 served as a positive toxicity control.

**Results:**

CS and PRO 2000 significantly protected mice from genital herpes following infection with a laboratory or clinical isolate of HSV-2 introduced in buffer (p<0.001). However, protection was reduced when virus was introduced in seminal plasma. Moreover, mice were significantly more susceptible to infection with low doses of HSV-2 when challenged 12 h after the 7th daily dose of CS or nonoxynol-9 (p<0.05). The increased susceptibility was associated with alterations in epithelial architecture.

**Conclusions:**

CS prevented genital herpes when present at the time of viral challenge, but increased the rate of infection when gel was applied daily for 7 days with a vaginal wash prior to viral inoculation. The findings presumably reflect altered epithelial architecture, which may have contributed to the trend towards increased HIV observed clinically.

## Introduction

Clinical trials of topical pre-exposure prophylaxis (PrEP) (also referred to as microbicides) for HIV prevention have yielded uniformly disappointing results, with the exception of the recent tenofovir gel trial [1]. Particularly concerning were the observations that some products not only failed to protect against infection, but were associated with higher rates of HIV acquisition in placebo-controlled efficacy trials [2]–[4]. While an increased risk of HIV following frequent exposure to a surfactant such as nonoxynol-9 (N-9) is not totally surprising, the observation in one of the trials that cellulose sulfate (CS) was associated with a trend towards higher rates of HIV infection compared to placebo (25 versus 16 new infections; relative risk = 1.61, p = 0.13) was unanticipated [2]. These results underscore the need to develop and validate more predictive preclinical biomarkers of safety and efficacy. This need will become even greater as partially protective topical and oral products are advanced into the clinic, rendering it more difficult and costly to conduct trials.

For a preclinical model to be valuable, it must be reflective of human disease and the results obtained with previously tested interventions should be consistent with the findings in clinical studies. Developing models of HIV transmission is particularly difficult because many of the key parameters are either unknown (e.g. inoculum dose) or difficult to simulate (e.g. effects of sex and semen on infection) [5]. Non-human primate (NHP) models provide important insights regarding HIV pathogenesis and potential efficacy of oral or topical PrEP [6], but so far have not proven to be consistently predictive of clinical outcomes [7]. For example, in a study conducted in rhesus macaques, none of the six animals treated intravaginally with 6% CS gel became infected compared to five of six macaques treated with hydroxyethylcellulose (HEC) placebo gel [8]. Similarly, Carraguard (a mixture of lambda and kappa-Carrageenan), which failed to prevent HIV infection in clinical trials [9], blocked vaginal transmission of RT-SHIV in macaque studies [10].

We previously published results with two potentially synergistic preclinical safety models: (i) a dual chamber culture model, which examines the impact products have on epithelial cell integrity and mediators of innate immunity [11]; and (ii) a murine model, which evaluates the effects of formulated gels on susceptibility to genital HSV-2 infection [12], [13]. We hypothesize that increased susceptiblity to HSV-2 in the murine model, whch may reflect changes in mucosal integrity and immune environment, will predict increased susceptibility to HIV infection. We applied these models to evaluate N-9, PRO 2000 and tenofovir [11]–[13]. The active pharmaceutical ingredients (APIs) were studied in the dual chamber model and the formulated gels in the mouse. Results obtained indicated that these models would have correctly predicted that 0.5% PRO 2000 and 1% tenofovir gel were safe.

We also tested CS as API in the dual chamber model [11] and found that single and repeated exposures to CS (100 µg/ml) triggered a decrease in transepithelial electrical resistance (TER) and an increase in HIV migration across the epithelial barrier, which resulted in infection of T cells cultured in the lower chamber. Building on this background, the current study was designed to test four blind-coded formulated gels (one of which was 6% CS) in murine models of efficacy and safety.

## Results

### Protection against genital herpes

**Figure 1:**
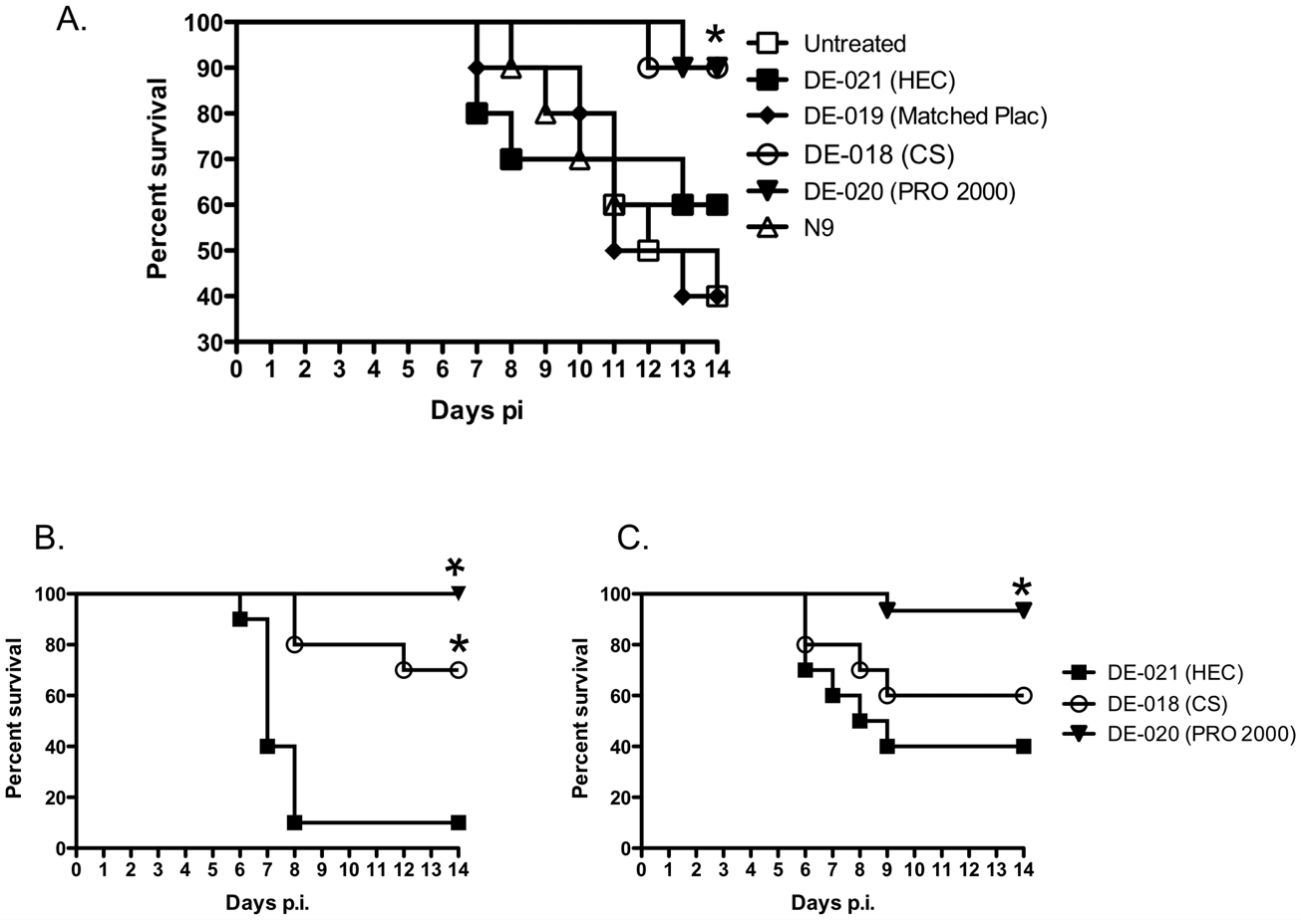
Cellulose sulfate and PRO 2000 gels protect mice from genital herpes, but the response is modulated when virus is introduced in seminal plasma. (**A**). Survival curves for mice pretreated with a single dose of each blinded gel or no gel (untreated) and challenged with an LD90 of HSV-29G) diluted in PBS. Survival curves for mice pretreated with a single dose of DE-021 (HEC), DE-018 (CS) or DE-020 (PRO 2000) and challenged with an LD90 of HSV-2(4674) diluted in PBS (**B**) or pooled human seminal plasma (**C**). Data were obtained from 2 independent experiments (10 mice per group). The asterisk denotes significant protection relative to HEC-treated mice (p<0.001 for virus introduced in PBS and p = 0.015 for virus introduced in seminal plasma).

Medroxyprogesterone acetate (MPA)-treated mice received a single application of each gel (or no treatment) and were then challenged 15 min later with ∼10^5^ pfu/mouse of HSV-2(G) delivered in PBS and monitored for signs of disease. This dose is lethal in 60–90% of mice. Two of the products, CS 6% gel and PRO 2000 2% gel, provided significant protection, keeping 90% of the animals alive 14 days post-inoculation, whereas the others (a placebo gel matched to the CS formulation and HEC, the universal placebo gel) had little or no protective effect (Fig. 1A, p<0.05). Notably, N-9 failed to protect against genital herpes. CS and PRO 2000 also provided significant protection relative to HEC against a clinical HSV-2 isolate (p = 0.0013 and p<0.0001, respectively) (Fig. 1B).

To further evaluate the active gels under conditions that more closely reflect what happens clinically, we also tested the activity when virus was introduced in seminal plasma. Delivering virus in seminal plasma reduced the effective inoculum such that a dose that killed 90% of HEC treated mice (LD90) when virus was introduced in PBS (Fig. 1B) was reduced to an LD60 (Fig. 1C). Under these experimental conditions, CS 6% gel no longer provided significant protection (p = 0.36), whereas the activity of PRO 2000 2% gel, though reduced, remained significant (p = 0.015). These findings are consistent with *in vitro* studies in which seminal plasma significantly reduced the antiviral activity of CS and PRO 2000 API by competitively blocking the binding of the polyanionic drugs to the viral envelope [14].

### Increased susceptibility to HSV-2 following exposure to gels

**Figure 2:**
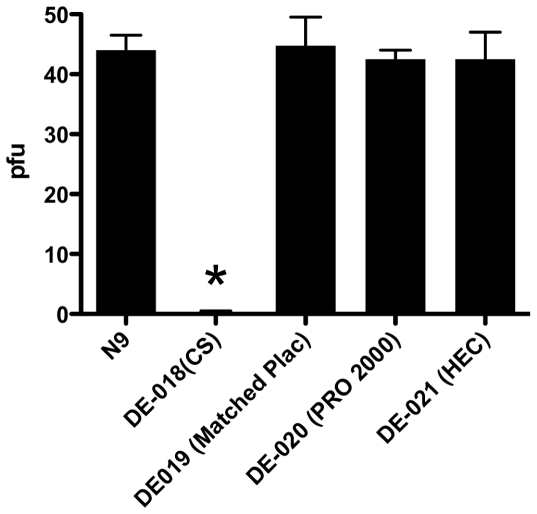
Vaginal washes from mice treated with cellulose sulfate 6% gel inhibit HSV-2 plaque formation. To remove any residual drug within the vaginal lumen prior to HSV-2 challenge in the safety studies, vaginal washes were obtained with 100 µl of saline. The vaginal washes were tested in a viral plaque assay. Results are means (SEM) from 2 independent experiments with pooled washes from 5 mice each; the asterisk denotes p<0.05.

To determine if any of the blinded gel products increased the susceptibility to low dose challenge with HSV-2, mice were treated once daily for seven consecutive days and then 12 h after the last gel application, vaginal washes were collected to remove any residual drug and subsequently assayed for anti-HSV activity (Fig. 2). There was sufficient drug within vaginal washes from mice that had received CS 6% gel, but not PRO 2000 2% gel, to inhibit HSV-2 infection in a plaque assay. This is in agreement with the bioadhesive properties of CS gel and its observed prolonged time of vaginal residence [15].

**Figure 3:**
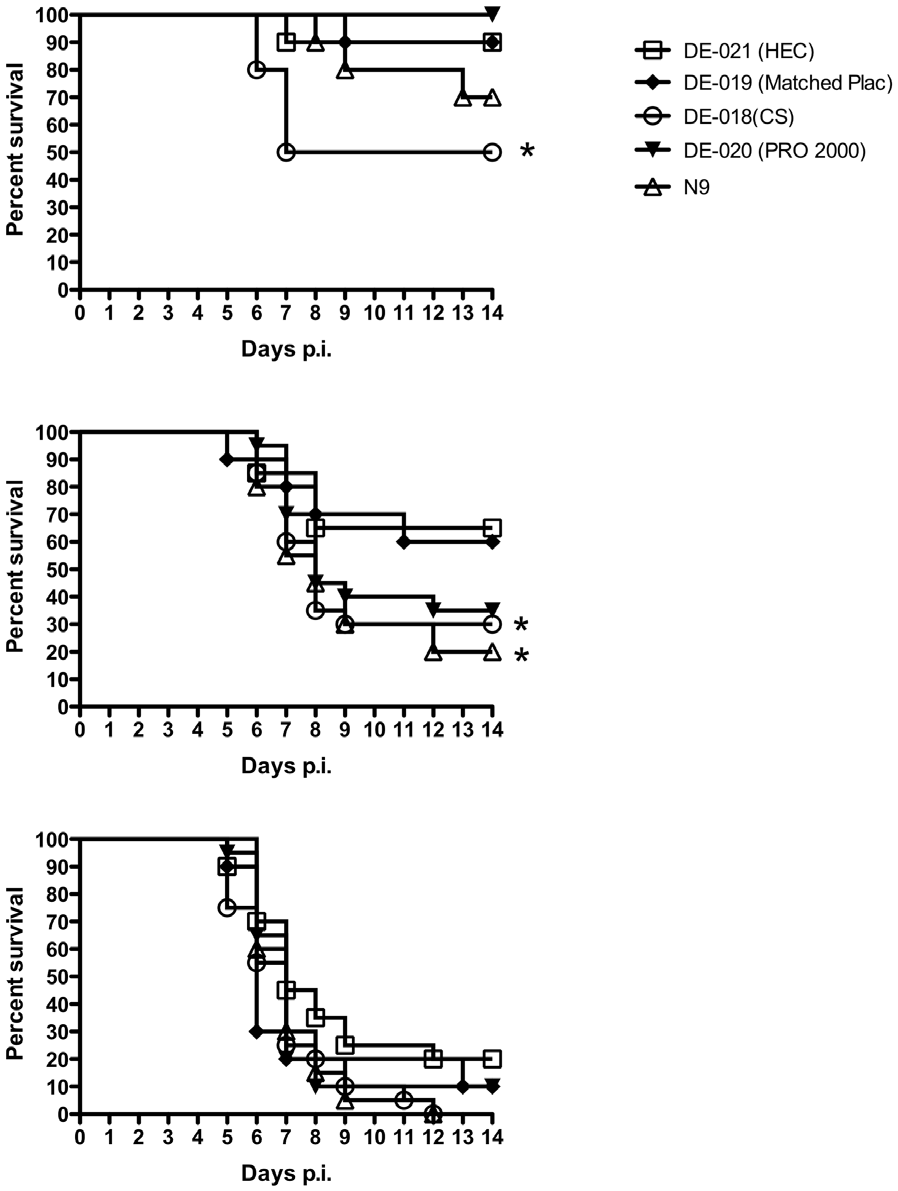
Increased susceptibility to HSV-2 following seven daily gel applications. Mice were challenged with ∼10^3^, 10^4^ or 10^5^ pfu/mouse of HSV-2(4674) (upper, middle and bottom panels, respectively), representing an LD10, LD40, and LD90 12 h after receiving the seventh daily dose of N-9 or coded gel products and after vaginal washing. Animals were observed daily for signs and symptoms of disease. Mice developing severe genital or neurological disease were euthanized. Results show percent survival pooled from at least 2 independent experiments (n = 20 mice/group). The asterisks denote p<0.05.

The mice were then challenged with serial dilutions of virus representing an LD10, LD40, and LD90 (doses at which 10, 40 and 90% of the population is expected to succumb to disease, respectively). Half of the mice pretreated with CS 6% gel succumbed to infection following challenge with an ∼LD10 compared to only 10% of HEC-treated mice (p = 0.05) (Fig. 3, upper panel). Similarly, 14 of 20 mice (70%) pretreated with CS succumbed to challenge with an ∼LD40 compared to 7/20 (35%) of mice treated with HEC (p = 0.03) (middle panel). Mice treated with PRO 2000 2% gel exhibited a non-significant trend toward increased susceptibility following challenge with an LD40 (p = 0.08), but no increase in susceptibility was observed following challenge with an LD10. There were no differences in survival curves between products following challenge with an LD90 (lower panel) and the majority of animals succumbed to disease with this high inoculum. N-9 significantly increased the susceptibility to genital disease after challenge with an LD40.

### Increased susceptibility reflects changes in epithelial architecture

**Figure 4:**
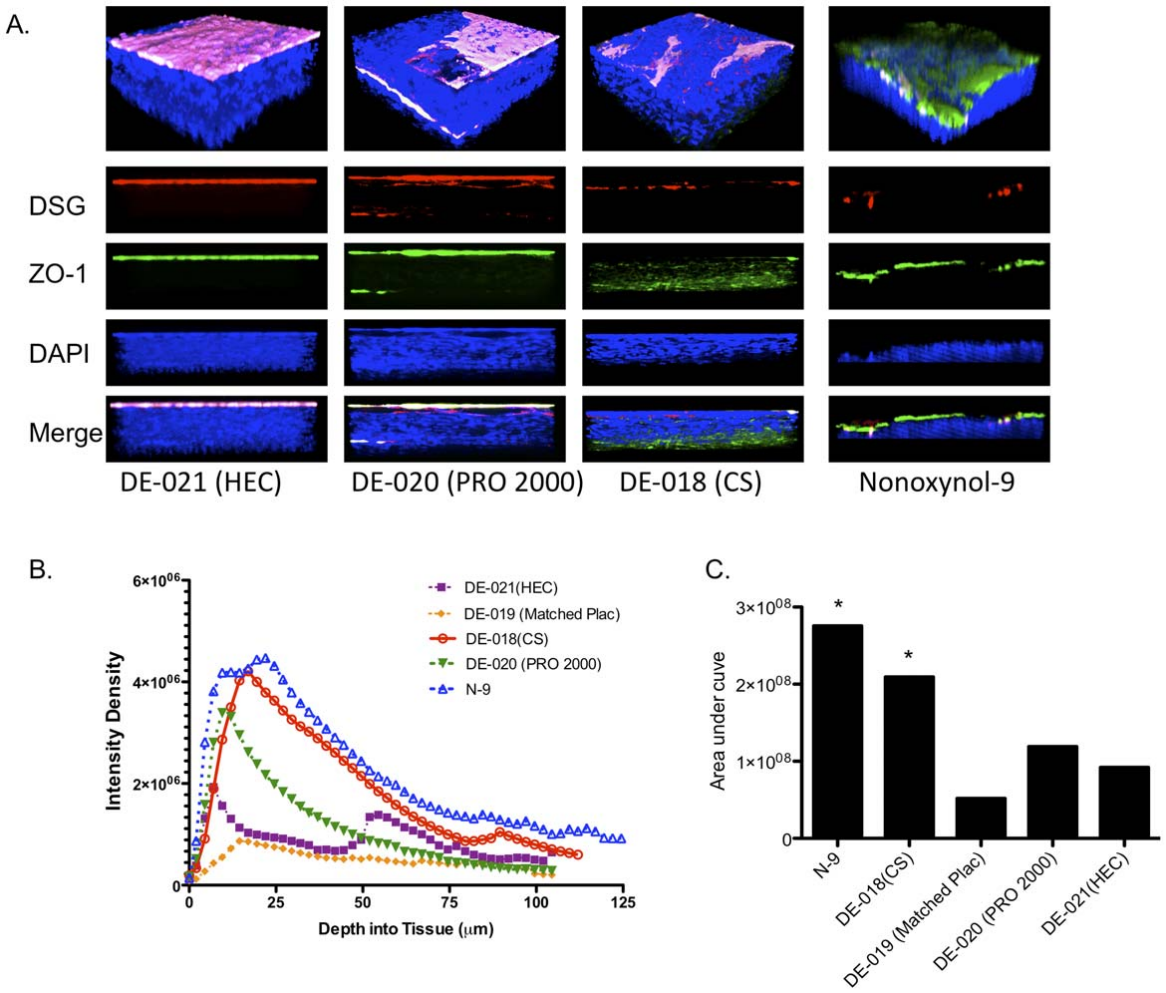
N-9 and cellulose sulfate causes disruption of the epithelium. Mice were treated daily for seven days with microbicides and twelve hours after the final application, the mice were sacrificed and the entire vaginal canal up to the uterine bifurcation was excised. The tissue was then fixed and stained with EZ-Link Sulfo-NHS-Biotin to detect the apical surface (magenta) and DAPI to detect nuclei (blue), ZO-1 (green) and desmoglein (DSG, red) and viewed by confocal microscopy. Representative 3-dimensionsal (upper panel) and xz (lower panels) images are shown (A). Images were taken from at least 3 animals per treatment group and at least 6 independent randomly selected images were acquired per animal. To assess whether disruption of the epithelial barrier promoted HSV migration through the tissue, mice were inoculated with HSV-2 12 hours after the seventh daily gel application and were then sacrificed four hours after infection. Tissues were stained for viral capsids with a mAb to VP16; phalloidin, which stains actin and was used to delineate the cytoplasm; and DAPI to identify nuclei. Individual Z slices were analyzed and intensity density of the fluorescence quantified. The results are means obtained from 8 independent randomly selected images (2 images per genital tract sample) (B) and the cumulative area under the curve is shown in C; asterisks indicate a significant increase in the area under the curve (p<0.001, ANOVA).

To determine if changes in the epithelium contributed to the increased susceptibility to HSV-2 in the mouse as had been observed in the dual chamber culture model [11], genital tract tissue was excised after seven daily doses of gel and evaluated by confocal microscopy. Images of the lower genital tract representative of results obtained from three mice per group are shown (Fig. 4A). The junctional proteins (zona occludens (ZO-1), green and desmoglein, red) were not readily detected in the 3-D images of HEC-treated tissue, reflecting compact epithelium with intact tight junctions. Treatment with N-9 led to thinning of the epithelium, loss of plasma membrane staining (magenta) and increased detection of ZO-1. Exposure to CS resulted in modest thinning of the epithelium, a reduction in plasma membrane staining, and increased visualization of desmoglein. In addition, in the xz images (lower panels), ZO-1 appeared to be mispolarized towards the basal side of the tissue and to be more dispersed compared to HEC-treated tissue. There was some loss of plasma membrane staining with a concomitant increase in the visualization of desmoglein in PRO 2000 treated tissue.

To address whether the changes in epithelial architecture facilitated the penetration of HSV into the epithelium, mice that had received seven daily doses of gel were challenged with HSV-2 and four hours after viral challenge, the animals were sacrificed and the presence of viral capsids was assessed by immunostaining with an antibody to the HSV capsid protein, VP16. The tissue was also stained with phalloidin (which stains actin) and DAPI to delineate the cytoplasm and nuclei, respectively. Z-stack images were obtained starting from the first detected fluorescent signal and continued in 0.5-micron increments and pixel intensities on axial images were quantified (Fig. 4B). Viral capsids extended more than 50 microns into the tissue following exposure to N-9 or CS and both products led to an increase in viral capsids detected within the tissue as evidenced by a significant increase in the area under the curve compared to tissue extracted from mice treated with either placebo gel or mice treated with PRO 2000 (Fig. 4B and 4C) (p<0.001, ANOVA).

### Exposure to gels triggers release of inflammatory cytokines and chemokines

**Figure 5:**
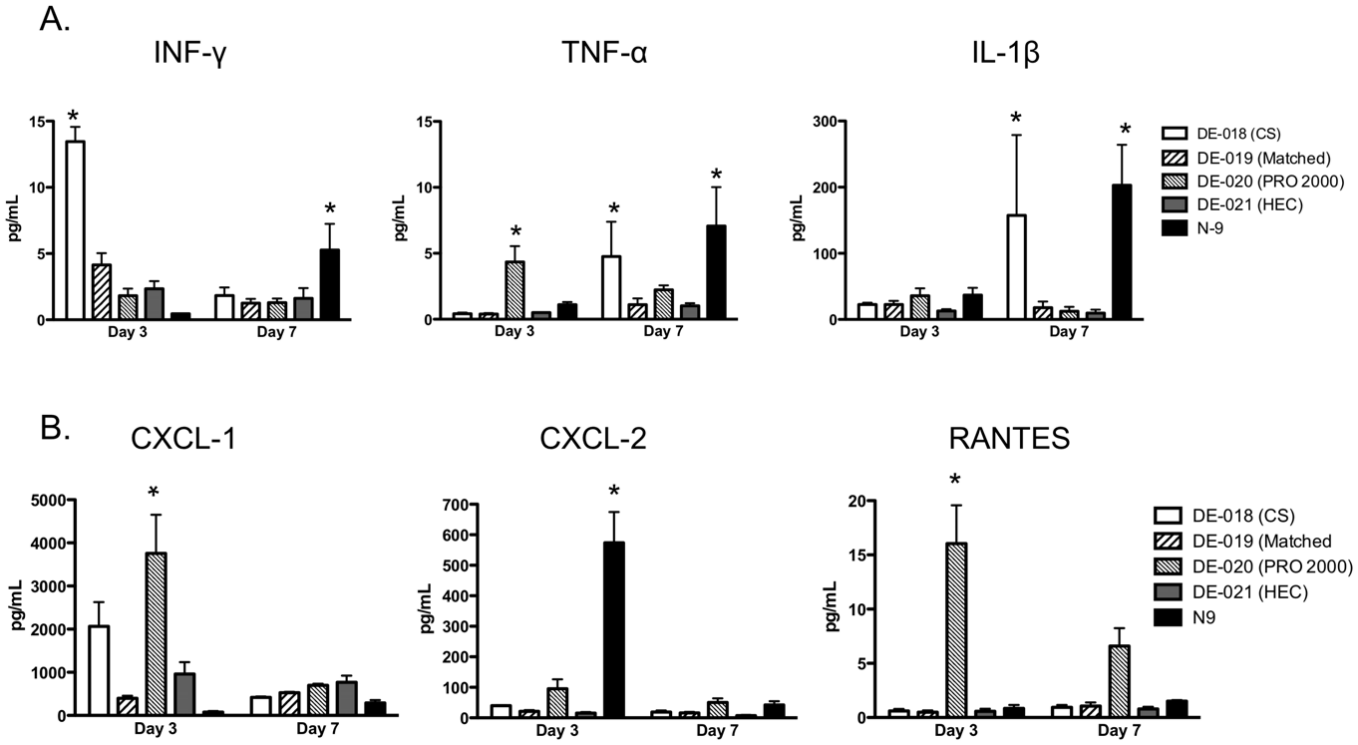
Changes in cytokines and chemokines in response to microbicides. The levels of chemokines and cytokines were measured in vaginal washes pooled from 5 mice by BioLuminex. Results are presented as mean ± SE values from six different pools (5 mice per pool) for washes obtained after three daily doses and from at least three pools for washes obtained after seven daily doses. The asterisks denote *p*<0.05 relative to HEC-treated mice.

Tight junctions are regulated in part by inflammatory cytokines such as IFN-γ, TNF-α, and IL-1β, which have been shown to trigger intestinal epithelial barrier dysfunction [16]–[18]. To determine whether the disruption in the epithelium was associated with increases in any of these cytokines, vaginal washes were analyzed for cytokine concentrations. Compared to mice treated with either placebo gel, N-9 induced a significant increase in all three inflammatory cytokines after seven doses, although no increases were observed after three daily doses. CS also induced significant increases in IFN-γ after the third gel application and TNF-α and IL-1β after seven daily doses. PRO 2000 induced only a transient increase in TNF-α after three doses (Fig. 5A).

Microbicides may also promote inflammation and recruitment of HIV target cells into the genital tract by triggering the release of chemokines. To explore this notion, vaginal washes were also analyzed for the presence of CXCL1 (KC), CXCL2 (MIP-2), and RANTES. Vaginal washes collected after three doses from mice treated with PRO 2000) had significantly more CXCL1 and RANTES and washes from mice treated with N-9 had higher levels of CXCL-2. There was also a non-significant increase in CXCL-1 in response to CS after three doses.

## Discussion

Precisely how HIV viral particles and/or infected cells traverse the epithelium lining the lower female reproductive tract to reach lamina propria and submucosal immune target cells and establish an infection is not fully understood. HIV transmission is increased when the integrity of the epithelium is impaired, as in the setting of ulcerations, inflammation or cervical ectopy [19]. Cervical ectopy, the presence of single-layered columnar epithelium outside the cervical os, an area normally lined by multilayered squamous epithelium, is more frequently present in adolescents and may be increased with hormonal contraception, pregnancy and cervical infections [20]. The link between HIV acquisition and conditions that disrupt the epithelial barrier highlights the need to develop and incorporate measurements of epithelial integrity in the assessment of topical product safety. However, there are no standardized validated methods to perform this evaluation.

Studies in NHP and early clinical safety studies have traditionally used colposcopy as a primary outcome measure. However, this approach has not always proven to be predictive of safety. For example, a standardized NHP model that incorporated colposcopy as a major indicator of safety concluded that 24 different products including several different formulations of N-9, 1% C31G, and 6% CS gel were safe [21]. Similarly, phase I clinical studies, which relied on colposcopy as primary endpoints, also failed to detect any potential safety concerns with these formulations [22]–[24]. In spite of their preclinically demonstrated antiviral activity, each of these products failed to protect women from HIV infection and was associated with at least a trend towards increased HIV acquisition in large-scale clinical effectiveness trials [2]–[4], [25].

More recently, optical coherence tomography (OCT) has been proposed as an alternative non-invasive method to assess the epithelium and has been evaluated in sheep and NHP studies with N-9 and benzalkonium chloride [26], [27]. OCT detected toxicities with these two surfactant products, but additional studies with non-surfactant compounds are needed to determine the predictive value of this methodology.

Results of the current study with coded gels suggest that a simple, relatively inexpensive murine model may provide a predictive measure of epithelial barrier integrity. The findings obtained parallel those in the dual-chamber cell culture model [11] and, most importantly, are consistent with the clinical effectiveness outcomes of three of the coded samples, 6% CS, 2% PRO 2000 and HEC (note that the placebo gel matched to CS was not evaluated in clinical trials). In this study, repeated intravaginal exposure of mice to 6% CS (and N-9) induced changes in the epithelial architecture that led to increased permeation of HSV viral particles through the epithelium. We speculate that a similar mechanism may have contributed to the trend towards increased HIV acquisition observed in one of the two CS trials [2]. In addition to the increased susceptibility to HSV, a modest but significant increase in IFN-γ, TNF-α, and IL-1β was detected in vaginal washes, which may have contributed to the epithelial barrier dysfunction. Phase I clinical studies, however, did not report an increase in cervicovaginal cytokines or chemokines upon repeated exposure to CS gel [22].

Consistent with studies performed with open-labeled PRO 2000 gel [13], modest changes in the epithelium and an increase in chemokines in vaginal washes, which were associated with a non-significant increase in the susceptibility to HSV, were induced by this product in the present study. These responses may have contributed to the lack of clinical efficacy of PRO 2000 gel [28]. Increasing the frequency or duration of exposure to products prior to viral challenge might unmask additional toxicities and requires further study.

Notably, mice are pretreated with MPA, which hormonally synchronizes the animals, thins the epithelium, and up-regulates the expression of the HSV co-receptor, nectin-1 [29], resulting in greater susceptibility to HSV infection. The increased susceptibility to genital herpes following MPA treatment parallels clinical findings of increased HIV risk in women treated with Depo-Provera® [30]. Thinning of the epithelium likely exacerbates the deleterious effects of compounds on epithelial integrity.

There are several limitations of the model that underscore the need to evaluate products in multiple systems to assess safety. For example, the mouse model does not provide an opportunity to evaluate the impact on vaginal flora, which also plays a key role in host defense. Recent data suggest that CS may modify epithelial-microflora interactions leading to disturbed vaginal microbial communities [31]. Another limitation is the species differences in the expression and regulation of soluble mucosal immune mediators such as defensins, which may impact the susceptibility to HIV and HSV infection [32]–[35]. Most importantly, the mouse is not susceptible to HIV infection rendering it necessary to rely on susceptibility to HSV as a surrogate. Humanized mouse models have been developed, but the extent to which these provide models of product safety is not yet known [36].

While the primary objective of these studies was to evaluate safety, we also assessed the efficacy of the coded samples when mice were challenged with a high dose of HSV-2 diluted either in PBS or in human seminal plasma. Both 6% CS and 2% PRO 2000 significantly protected mice from disease when either a laboratory strain or clinical isolate was introduced in PBS. However, the protective effect was reduced when virus was diluted directly into human seminal plasma. These findings are consistent with a recently completed postcoital clinical study with PRO 2000 [37] and likely reflect competition between seminal plasma proteins and polyanionic drugs for binding sites on the viral envelope [14]. We speculate that the reduced antiviral activity of CS and PRO 2000 in the presence of semen, combined with impaired epithelial architecture, overcame the anti-HIV efficacy of these products.

Results of the current studies indicate that these simple and relatively inexpensive murine models should be considered early in the preclinical development of of next generation candidate topical PrEP. While the efficacy of HIV-specific products cannot be tested in mice, the findings support the need to evaluate efficacy in alternative in vitro or animal models where HIV is introduced in semen prior to initiating clinical trials. Validated biomarkers of efficacy and safety will become of even greater importance as we strive to improve upon the encouraging results obtained with topical tenofovir.

## Materials and Methods

### Cells and viruses

CaSki (human cervical) and Vero (monkey kidney) epithelial cell lines were obtained from the American Tissue Culture Collection (ATCC); human keratinocytes (HaCAT) were obtained from David Johnson (Oregon Health & Sciences University, Portland, OR). All cells were grown and maintained in Dulbecco's modified Eagles medium (DMEM) supplemented with 10% fetal bovine serum (FBS). HSV-2(G), originally isolated from a genital lesion [38], was grown on Vero cells and HSV-2(4674), a clinical isolate obtained from the clinical virology laboratory at Montefiore Hospital, was propagated on HaCAT cells.

### Gels

Four coded gels were provided by CONRAD (DE8-018, DE8-019, DE8-020, and DE8-021) and upon locking the data, were identified as 6% CS, a placebo-matched control, 2% PRO 2000, and HEC-based universal placebo, respectively. Encare, which contains 4% N-9 (Blairex Laboratories, Inc., Columbus, IN), was included as an unblinded positive control for toxicity.

### Murine model

Studies were conducted with approval of the Albert Einstein College of Medicine Institutional Animal Care and Use Committee. To assess whether gels prevented genital herpes (efficacy), female BALB/c mice (7–10 weeks) were pretreated subcutaneously with 2.5 mg of MPA (Sicor Pharmaceuticals, Irvine, CA, USA) and five days later were inoculated intravaginally with 20 µl of each blinded gel or N-9. Fifteen minutes later, mice were challenged with 30 µl of virus (equivalent to ∼10^5^ plaque forming units (pfu)). The virus was diluted either in phosphate buffered saline (PBS) or in pooled human seminal plasma (Lee BioSolutions, St Louis, MO). The mice were evaluated for 14 days for evidence of erythema, edema, genital ulcers, hair loss around the perineum, urinary or fecal retention and hind-limb paralysis and were euthanized if symptoms of severe genital tract or neurologic disease developed [39].

To assess whether exposure to gel increased the susceptibility to infection, 40 µl of each gel was delivered intravaginally daily for seven days to MPA-treated mice. Approximately 12 hours after the seventh application, vaginal fluid was collected by washing with 100 µl of normal saline and then groups of five mice were inoculated with HSV-2 equivalent to approximately 10^3^, 10^4^ and 10^5^ pfu per mouse. Additional mice were treated daily for 7 days with each gel (first dose delivered on Day 0) and vaginal washes (100 µl of normal saline) were collected for detection of cytokines and chemokines from groups of five mice on days 0, 3 (prior to vaginal dosing) and on day 7. Mice from each treatment group (n = 3–5) were sacrificed at different times and genital tract tissue excised and processed for confocal microscopy.

### Plaque assays

CaSki cells were grown in 24-well plates and were then inoculated with 10^3^ and 10^4^ pfu/ml of HSV-2(G) that had been mixed 1∶1 with vaginal washes pooled from 5 mice per treatment arm. After incubation for 1 h, the cells were washed and overlaid with methylcellulose (0.5% methylcellulose dissolved in medium 199 supplemented with 1% heat-inactivated FBS). Viral plaques were counted by a “black-plaque” immunoassay 48 h after infection using a rabbit polyclonal anti-HSV antibody (1∶500; Genway Biotech, Inc., San Diego, CA) and a goat anti-rabbit IgG horseradish peroxidase conjugate (1∶500; Bio-Rad Laboratories, Hercules, CA) [40].

### Confocal microscopy

Genital tract tissue was excised and processed using previously detailed methods [13]. The plasma membranes were stained with EZ-link sulfosuccinimidobiotin reagent (EZ-link, 1∶1,000; Pierce, Rockford, IL, USA), which reacts with primary amines on cell surface proteins. Non-specific antibody binding sites were blocked by overnight incubation at 4°C with PBS containing 10% goat serum and 1% bovine serum albumin. Subsequently, bound EZ-link was detected by treatment with streptavidin conjugated to Alexa Fluor 647 (1∶1,000); tight junctions were detected with rabbit anti-zona occludens protein 1 (ZO-1; 1∶500) and Alexa Fluor 488-conjugated secondary antibody (1∶1,000), and adherens junctions were detected with mouse anti-desmoglein-1 (1∶500) and Alexa Fluor 555-conjugated secondary antibody (1∶1,000) (all antibodies from Invitrogen, Carlsbad, CA, USA). Nuclei were detected by staining with 4′,6′-diamidino-2-phenylindole nucleic acid stain (DAPI; Molecular Probes, Inc.; 1∶2000 dilution; Eugene, OR).

To examine the extent of HSV penetration through the tissue, tissue was excised four hours after HSV challenge and incubated with mouse anti-viral capsid protein,VP16 (1∶500; Santa Cruz Biotechnology, Santa Cruz, CA) and Alexa Fluor-488 secondary antibody. F-actin was stained with Alexa Fluor 555 Phalloidin (Invitrogen; 1∶40; Carlsbad, CA) and nuclei were stained with DAPI. The tissue was mounted on glass slides using ProLong Gold Antifade reagent (Invitrogen) and images were obtained on a Leica SP5 AOBS confocal microscope (63x objective; three-dimensional (3-D) composite images were created from Z-stack images (Volocity Software). Individual Z slices were analyzed using Image J software to quantify the fluorescence.

### Cytokine and chemokine analyses

Protease inhibitors (Complete Protease Inhibitor Cocktail; Roche Applied Science, Indianapolis, IN) were added to each pooled vaginal wash sample before centrifugation at 210 *g* for 10 min at 4°C. The supernatants were stored at −80°C and assayed for cytokines and chemokines using the Fluorokine MultiAnalyte profiling system (R&D Systems, Minneapolis, MN, USA), measured with a Bioluminex 100 system (Bioluminex, Austin, TX) and analyzed with StarStation (version 2.0; Applied Cytometry Systems, Sheffield, UK).

### Statistical analyses

Kaplan-Meier survival curves were assessed by log-rank test and cytokines and chemokines were analyzed by one-way analysis of variance (ANOVA) using Prism software (version 5; GraphPad).
